# Smell and Stress Response in the Brain: Review of the Connection between Chemistry and Neuropharmacology

**DOI:** 10.3390/molecules26092571

**Published:** 2021-04-28

**Authors:** Yoshinori Masuo, Tadaaki Satou, Hiroaki Takemoto, Kazuo Koike

**Affiliations:** 1 Laboratory of Neuroscience, Department of Biology, Faculty of Science, Toho University, 2-2-1 Miyama, Funabashi, Chiba 274-8510, Japan; 2 Department of Pharmacognosy, Faculty of Pharmaceutical Sciences, International University of Health and Welfare, 2600-1 Kitakanemaru, Ohtawara, Tochigi 324-8501, Japan; tsatou@iuhw.ac.jp; 3 Department of Pharmacognosy, Faculty of Pharmaceutical Sciences, Toho University, 2-2-1 Miyama, Funabashi, Chiba 274-8510, Japan; hiroaki.takemoto@phar.toho-u.ac.jp (H.T.); koike@phar.toho-u.ac.jp (K.K.)

**Keywords:** stress, smell, aroma, olfaction, brain, anxiety, fatigue, human, rat, mouse

## Abstract

The stress response in the brain is not fully understood, although stress is one of the risk factors for developing mental disorders. On the other hand, the stimulation of the olfactory system can influence stress levels, and a certain smell has been empirically known to have a stress-suppressing effect, indeed. In this review, we first outline what stress is and previous studies on stress-responsive biomarkers (stress markers) in the brain. Subsequently, we confirm the olfactory system and review previous studies on the relationship between smell and stress response by species, such as humans, rats, and mice. Numerous studies demonstrated the stress-suppressing effects of aroma. There are also investigations showing the effects of odor that induce stress in experimental animals. In addition, we introduce recent studies on the effects of aroma of coffee beans and essential oils, such as lavender, cypress, α-pinene, and thyme linalool on the behavior and the expression of stress marker candidates in the brain. The transfer of volatile components into the brain is also discussed while using the results of thyme linalool as an example. These studies may provide a good opportunity to connect chemical research at the molecular level with neuropharmacological approaches in the future.

## 1. Introduction

Stress is a risk factor in the development of mental disorders. Anxiety regarding the future and life that dominates society as a whole has become a major issue for mental health. A large number of researchers have studied the effects of stress on animals, including humans, and they have demonstrated changes in the endocrine system, especially via the hypothalamic-pituitary-adrenal (HPA) axis [1,2]. Although the stress response of the brain and the onset mechanism of mental disorders have not yet been elucidated, the suppression of stress is important in preventing the onset of mental disorders. It has been empirically known from ancient times that the aroma and fragrance have the effect of suppressing stress. Human beings have become more dependent on vision than other species in the process of evolution, and the olfactory system is thought to be degenerated [3]. However, humans are also a member of mammals, and the olfaction can affect emotions, higher functions, and the autonomic nervous system [4,5]. Indeed, the relationship between stress and olfactory memory has been suggested in humans [6,7]. The subjective discomfort of odors *per se* provokes the stress response in emotion and the sympathetic nervous system in humans [8]. On the contrary, certain smells suppress stress level [9], which is supported by the evidence showing the effects of exogenous volatile components on the brain [10].

There are a wide range of words regarding smell, which are not academic terms and the difference in meaning is not clear. For this review, the authors have endeavored to be based on the Cambridge Advanced Learner’s Dictionary, 4th Edition, Cambridge University Press, as much as possible. Specifically, “smell” is the quality something has that you notice by using your nose. “Fragrance” and “scent” represent a good smell. “Aroma” expresses a nice smell that usually comes from food or drink. However, the “aroma” used in aromatherapy has the meaning of smell and fragrance. “Odor” means a smell, often one that is unpleasant. Although these different expressions are intricate, the target chemicals can sometimes be the same. Even if certain smell is pleasant at low concentrations, it may turn into unpleasant at high concentrations. It is also possible that a high dose of volatile compound may have toxicity. However, the dose-response of volatile components has often been unclear in the literature. In addition, the effects of volatile compounds may appear not only via the olfactory pathway but also via the transport of molecules to the brain (brain transferability), which makes it difficult to study the effects of smell on the brain.

This review first outlines what stress is and previous studies on stress-responsive biomarkers (stress markers). Subsequently, we would like to focus on the relationship between the smell and the stress response in the brain. Research on stress-suppressing aromas is conspicuous, but some odors induce stress, which, in turn, enables the survival of the species. Moreover, we introduce recent animal studies showing the effects of aroma on the behavior and expression levels of stress marker candidates in the brain as examples of coffee bean aroma and fragrance of essential oils, such as lavender, cypress, α-pinene, and thyme linalool. In addition, here we discuss the permeability of volatile components to the brain by taking thyme linalool as an example.

## 2. Stress Evaluation in Mammals

### 2.1. Stress

A short article that was published by Hans Selye in the Nature [11] is well known as “Stress Theory”. He calls the state in which the living body is distorted by external stimuli, such as “stress”, and the stimulus that causes stress as “stressor”. There are four kinds of stressors, such as physical, chemical, biological, and mental stressors. When exposed to a stressor, the organism not only specifically responds to the type of stimulus, but also to a series of non-specific reactions that are independent of the type of stimulus. This non-specific reaction is called stress reaction. The word “stress” that is commonly used today means “stressor”. It may be of interest that stress is divided into “eustress” and “distress”, depending on whether it has a positive or negative effect on the organism. The balance between these two affects the state of health.

**Figure 1:**
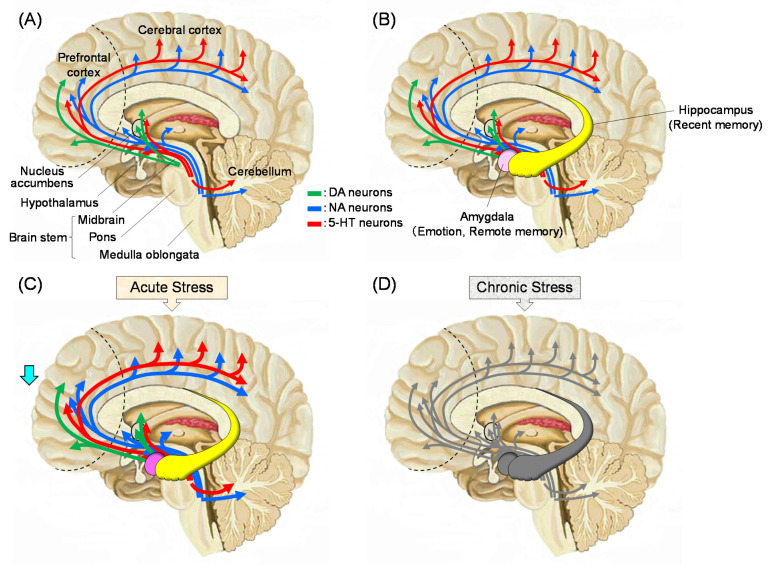
Sagittal sections cut in the middle of the human brain. (**A**): The main pathways of monoaminergic neurons. Dopamine (DA) neurons are highly located in the midbrain (substantia nigra pars compacta and ventral tegmental area). The nigro-striatal pathway is mainly related to the motor activity. The mesolimbic pathway to the nucleus accumbens, frontal cortex and amygdala is involved in emotions, which is called the reward system. Noradrenaline (NA) and serotonin (5-HT) neurons in the brain stem (locus coeruleus and raphe nucleus, respectively) project extensively. (**B**): The brain with the limbic system. The hippocampus plays important roles in recent memory. The amygdala is involved in emotion and remote memory, which is related to fear memory. (**C**): Effects of acute stress. Stress activates the amygdala and the release of neurotransmitters from monoaminergic neurons. The sympathetic nervous system becomes dominant over the parasympathetic nervous system. (**D**): Effects of chronic stress. Neurons are then depleted of neurotransmitters and become less active. Cortisol level enhanced by stress in the blood reduces brain-derived neurotrophic factor (BDNF) levels in the brain, which causes neurodegeneration.

Stress causes alterations in the HPA axis and it affects the function of whole body [2,3]. These changes lead to modifications in the brain with negative feedback of the endocrine system [12]. The effects of stress include increased levels of cortisol in human blood (corticosterone in rodents [13]) and decreased function of the prefrontal cortex. Figure 1 summarizes what is currently known about the stress response in the brain. The functions of monoaminergic neurons, such as dopamine (DA), noradrenaline (NA), and serotonin (5-HT), which are projected to the prefrontal cortex, are enhanced early after stress exposure. This reaction is the so-called “fight or flight response”, in which the function of the prefrontal cortex is relatively low. Afterwards, monoamine neurotransmitters are depleted, and the neuronal functions becomes lowered. Stress-induced decline in limbic DAergic neurons (projection from the ventral tegmental area to the nucleus accumbens, frontal cortex, and amygdala, the so-called reward system leads to a loss of pleasure. Stress levels are strongly suggested to be related to reduced activities of 5-HTergic neurons in the amygdala and hippocampus [14]. Moreover, an increase in cortisol concentration due to stress causes a decrease in the expression of brain-derived neurotrophic factor (BDNF) in the brain, which results in neuronal cell death, especially in the fragile hippocampus. Thus, the brain function deteriorates, which leads to an increase in the incidence of mental disorders, such as depression [15]. 

Regarding changes in the brain that are related to stress and olfaction, corticotropin releasing factor (CRF)-containing neurons from the central amygdala [16,17] projecting to the bed nucleus of the stria terminalis [16,18] are suggested to be involved in the stress response. This projection is highly related to γ-aminobutyric acid (GABA) containing neurons [18]. Chronic stress in adulthood causes alterations in the expression of glucocorticoid receptors and CRF in the central amygdala, which results in visceral hypersensitivity [19]. The NA neurons in the locus coeruleus have been implicated in cognitive aspects of the stress response, in part through its regulation by opioid and the stress-related neuropeptide, CRF [20]. Moreover, somatostatin- and neuropeptide Y-containing neurons in the amygdala [21] and hypothalamus [22] may respond to stress.

### 2.2. Stress-Responsive Biomarkers (Stress Markers)

Stress markers will enable an objective understanding of stress levels in pre-disorder and contribute to early detection and the prevention of psychiatric and neurodegenerative disorders, including depression and dementia. In a relatively early paper [1], the use of blood plasma was a major material for measuring glucocorticoid hormones cortisol or corticosterone). Alternative methods were developed, such as the measurement of corticosteroids in saliva, urine, or feces to overcome the stress that is induced by blood sampling itself. Concerning the effects of chronic stress, hormonal secretions are usually unchanged, but functional changes are observed at several levels of the system, including the sensitization of the adrenal cortex to ACTH and the resistance of the HPA axis to feedback inhibition by corticosteroids (dexamethasone suppression test). BDNF, which is expressed in the brain and blood, has been demonstrated as a stress marker [22]. Cortisol or corticosterone levels in the blood elevated by stress provide feedback to the brain. In the hippocampus, which is a region vulnerable to stress [23], a decrement in the expression of BDNF causes neuronal cell death, resulting in cognitive decline. Because BDNF concentration in the blood of patients with major depressive disorder is lower than that of healthy subjects under certain conditions [15], BDNF is expected to be a candidate for a biomarker for a diagnosis of depression.

Salivary biomarkers are readily accessible and easily obtained. α-Amylase in saliva has been suggested as a sensitive biomarker for stress-related changes in the body reflecting the activity of the sympathetic nervous system [8,24]. Salivary stress markers are suggested to include α-amylase, cortisol, chromogranin A (CgA), and immunoglobulin A (IgA) [25]. Farah et al. [26] reviewed the literature on salivary biomarkers for diagnosing and monitoring psychiatric and neurodegenerative disorders, such as autism and Alzheimer’s, Parkinson’s, and Huntington’s disease. These biomarker candidates are amyloid β peptide, tau protein, lactoferrin, α-synuclein, DJ-1 protein, chromogranin A, huntingtin protein, DNA methylation disruptions, and micro-RNA profiles. Giacomello et al. [27] described that cortisol may be the currently best evaluated stress marker, and NA or its metabolite, 3-methoxy-4-hydroxyphenylglycol, is also considered to be a good stress marker. They cited, as other stress marker candidates, testosterone, dehydroepiandrosterone (DHEA) and its sulfonated analogue DHEA-S, α-amylase, secretory immunoglobulin A, and chromogranin A. Quite recently, Chojnowska et al. [28] suggested, in their review article, that promising salivary biomarkers of stress, anxiety, or depression are cortisol, immunoglobulin A (IgA), lysozyme, melatonin, α-amylase, chromogranin A, and fibroblast growth factor 2 (FGF-2). Among these, they mentioned cortisol, lysozyme, α-amylase, and chromogranin A as the most valuable potential markers of stress. Cortisol and melatonin are also suggested to be useful for the differentiation of depression from stress.

### 2.3. Recent Exploratory Research on Novel Stress Marker Candidates

Cortisol, α-amylase, chromogranin A, etc. have been established as stress markers, as mentioned above. Although these factors are effective for an evaluation of acute stress in healthy subjects, it is not suitable for the purpose of grasping changes in brain function due to chronic stress. In addition, many studies conducted around the world aim to find novel biomarkers of disorders, contributing to post-onset diagnostic methods. The authors have loaded various types of stress on experimental animals and they have found a group of new stress marker candidates in the brain. We have mainly studied developmental disorders due to chemical stress [29,30,31], schizophrenia [32], and chronic alcoholism [33]. As for chemical stress, we have been studying the effects of environmental chemical substances [34,35,36] and the effects of radiation as physical stress [37,38]. Furthermore, the effects of restraint, disturbance of the light-dark cycle, and water immersion (insomnia due to an unpleasant environment) were investigated in order to clarify the process from stress to the onset of depression.

OMICS analysis was applied to screen for novel stress markers. Marker candidate genes were identified by DNA array analysis, and the proteins by two-dimensional electrophoresis and mass spectrometry. We have found several new stress marker candidate genes that show changes in expression in both the brain and blood. Alterations in the expression of biochemical parameters in the brain are unlikely to be immediately reflected in peripheral blood, and the mechanisms that connect changes in the brain and blood should be further clarified. At least, the, stress response in the brain can be extrapolated by the results of blood tests if the correlation is established between changes in the brain and blood. By establishing an extrapolation method for changes in the brain by measuring blood stress markers, it will be possible to objectively grasp the stress level in the future. This will contribute to the prevention of the onset of mental disorders and the evaluation of therapeutic effects.

## 3. Olfaction and Stress Response in the Brain

### 3.1. Olfactory System

**Figure 2:**
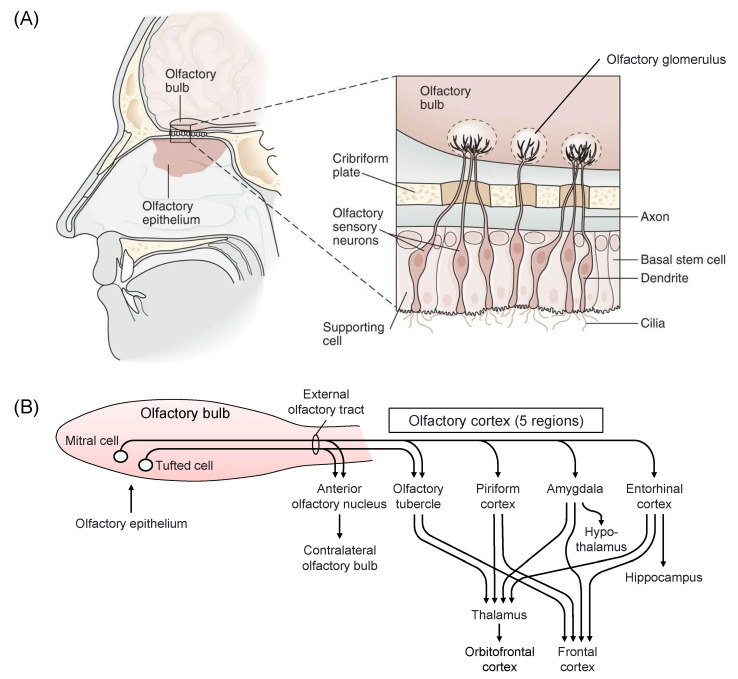
The human olfactory system. (**A**): Structure of the olfactory epithelium. There are 50 million olfactory sensory neurons in the nasal olfactory epithelium. Each neuron expresses one type of olfactory receptors on the cilia protruding toward the nasal cavity. From the other side of the neuron, an axon projects into the olfactory glomerulus in the olfactory bulb, where it synapses with the dendrites of the mitral and tufted cells. (**B**): Diagram of the olfactory pathway. Mitral cells project to the five regions, and tufted cells project to two regions in the olfactory cortex. Four regions connect to the thalamus and frontal cortex. Conscious discrimination of smell depends on the orbitofrontal and frontal cortices. Emotive aspects of olfaction derive from limbic projections (amygdala and hypothalamus). Thus, olfactory information may be closely related to memory and emotion, which affects stress response. This figure is derived from Kandel, E.R.; Schwartz, J.H.; Jessell, T.M. (Eds) Principles of Neural Science, 4th ed. McGraw-Hill, New York, NY, USA, 2000 [39] with a copyright permission.

The olfactory pathway has been clarified relatively recently [39] (Figure 2). The existence of a unique subsystem is suggested in the main mammalian olfactory system. This system may be comprised of sensory neurons expressing odorant receptors (ORs) of the OR37 subfamily. OR37 receptor subtypes A, B, and C were activated by the long-chain aliphatic aldehydes pentadecanal, hexadecanal, and heptadecanal, respectively, and the OR37 system was suggested to mediate social buffering [40]. Scientific knowledge with the keywords, “olfaction”, “stress”, and “brain” has been accumulated. Olfaction, which is the oldest phylogenetic sensation, is characterized by a unique intimacy with the emotion system, and certain smell causes stress and others suppress stress. The olfactory system has been suggested to be dynamic, with neuroplasticity in olfactory perception and memory [41]. Smell perception may be strongly related to functions of amygdala and hippocampus [41]. Indeed, human and animal studies suggest that olfaction may be modulated by experience and/or physical state, and some smells induce emotion and lead to the recall of emotional memories [42,43]. Moreover, it was demonstrated that stress affects smell recognition, including the development of olfactory system [44,45,46]. Numerous papers reported the effects of odors of mammalian endogenous substances as predator odor stress [47,48]. Chronic stress leads to long-term deficits in odor-driven behaviors that are critical for species survival and reproduction, and to neuroplastic changes in the nucleus of the lateral olfactory tract [49]. Social isolation has been suggested to modulate olfaction and social memory persistence, probably by independent mechanisms [50]. Odor may provoke a physiological response in the autonomic nervous system. An unpleasant aversive odor causes non-invasive stress, while a pleasant smell promotes healing and relaxation in mammals [51]. Moreover, it was demonstrated that the sweat odor of patients with panic disorder was positively correlated with the severity of psychopathology [52]. 

### 3.2. Stress-Inducing Smell

In humans, it was demonstrated that the secretion of salivary α-amylase was increased upon exposure to malodors, which suggested that subjective unpleasantness of odors can induce the stress response [8]. However, any other human studies on the induction of stress by specific odors were not found in the literature. In fact, experiments investigating the effects of certain odors that induce stress on humans must be ethically difficult to perform. In animal studies, prenatal stress can interfere with the development of the olfactory system during the prenatal period, leading to neurogenesis and altered olfactory capacity in the adulthood [53]. Maternal odor affects child development on the cognitive function [54] and in the social buffering, a phenomenon that social supports attenuate stress response [55].

A large number of studies were carried out on the effects of endogenous odor of animals, which suggested that predator-emitted odor molecules are stressors for prey animals. It is really important for species conservation to identify the dangers to life among the volatile components in the environment. These studies examined the stress response that odors cause in rodents as stressors. It has been suggested that the predator odors are a strong stressor [5], which leads to post-traumatic stress disorder (PTSD) [4]. Indeed, stress due to predator odor activates the HPA axis [56]. Gu et al. [57] demonstrated, in the Brandt’s vole, that maternal stress induced by predator odors during gestation restrained the growth of female offspring and increased the duration of exploring and foraging behaviors of male offspring by regulating the HPA axis. Therefore, the offspring became less sensitive to unfamiliar environments and more likely to be prey. As a predator’s stress on rodents, many researchers used cats [58,59,60], hamsters [61], guinea pigs [61], and ferrets [16,21,62]. In the case of cat odors, the effects of its urine [17,56,63,64] and feces [65] were examined. The urine of fox [66,67] and coyote [66] was also tested and it suggested a role for adolescent stress as a risk factor for psychosis, particularly in those with a heritable predisposition [67].

In view of molecules that stimulate the olfaction, 2,5-dihydro-2,4,5-trimethylthiazoline (TMT) is the main component of fox feces. Therefore, TMT is a predator odor for rodents, and it causes PTSD as a serious stressor [13,18,68,69,70,71,72,73,74]. Miyazono et al. [75] found that a mixture of three pyrazine analogues (P-mix) present at high levels in wolf urine induced fear-related responses in mice, which was attenuated by etizolam, an anxiolytic. On the other hand, stressed rats release odor cues, which allows other rats to avoid danger. Inflammatory cytokine responses in the brain can mediate such stress-induced chemical signals [76]. Moreover, Inagaki et al. [77] reported the identification of a pheromone that induces anxiety in rats. Stressed rats release a specific odor into the air, and this odor enhances the anxiety levels in other rats. Therefore, anxiety-causing molecules may be present in the stress-related odorants, which may be useful in understanding mammalian chemical communication. They suggested that this odor may be involved in 4-methylpentanal and hexanal. On the response to odor stress, gender differences are pointed out [13,18,70,78,79]. Harris et al. [80] demonstrated the social defeat paradigm that works in female mice. If male odorants are applied to female mice, females repeatedly undergo social defeat stress by aggressive behavior of resident males. Subsequently, females develop social avoidance, reduce the function of reward system, and enhance anxiety level. Such differences are also reported in social play behavior [62].

Concerning brain regions that are responsible for odor stress, Kondoh et al. [48] suggest that the amygdalo-piriform transition area, a small region of the olfactory cortex, may play a key part in the hormonal component of the instinctive fear response to volatile predator scents. The relationship between the olfaction and the amygdala and hippocampus has been suggested. Fujimoto and Aou [68] observed that prenatal treatment with low-level bisphenol A, one of environmental chemicals, impaired gender-specific behavior, enhanced depression-like behavior, and augmented behavioral responses to predator odor. Interestingly, mild exposure to a predator odor during hippocampus-dependent learning leads to increasing performance through the co-activation of the amygdala, which is probably by a stress mediated mechanism [69]. Moreover, it was shown that trpa1-mediated nociception plays an important role in the innate fear/defense behavior that is caused by predator odors [74].

### 3.3. Stress-Suppressing Smell

Many studies mainly demonstrated the stress-suppressing effect of essential oils. Table 1 summarizes the literature. The articles are introduced separately for humans, rats, and mice.

#### 3.3.1. Effect on Humans

Even at non-life-threatening levels, chronic stress is a risk factor for the development of various mental disorders, and it is desirable to control the stress levels. The stress-suppressing effects of certain smell have been recognized, and the effects of fragrances on human psychophysiological activity have been studied for a long time [10]. Granqvist et al. [9] pointed out that, in stressful situations, access to adult attachment figures (e.g., romantic partners) is an important means by which adults regulate stress responses. They observed in humans that partner body odor reduced subjective discomfort during a stressful event, as compared with the odor of oneself, which suggests that partner odor is a scent of security, especially for attachment-secure adults. Yoto et al. [81] investigated the alleviating effect of black tea aroma on physical and psychological stress in humans that is induced by the Uchida–Kraepelin test, based on salivary chromogranin-A (CgA) levels as a stress marker and subjective evaluations. It was observed that inhaling black tea aroma can reduce stress levels, and Darjeeling tea aroma tends to improve mood before exposure to mental stress.

Several lines of evidence in human studies suggest the stress suppression effects of essential oils, such as lavender [82,83,84,85,86], rose [91], rosemary [86], bergamot [93,94,95], chamomile [83], grapefruit [97], and neroli (*Citrus aurantium* L. var. *amara*) obtained from the flowers of bitter orange [98]. Stress attenuating effects are also observed in essential oils that are derived from trees, such as cypress [99] and cedar [102]. The majority of human studies relate to assessing the effects of essential oils in clinical applications. Essential oils can reduce the stress levels at various levels. In human studies, psychiatric interviews are possible, and conditions, such as mood, can be evaluated. It is also possible to measure vital signs such as body temperature, blood pressure, and heart rate to evaluate the sympathetic nerve activity, and the concentration of stress hormones, such as cortisol in blood and stress markers in saliva. Therefore, the data showing that the aroma of essential oils suppressed the changes in these parameters caused by stress must be the evidence that the smell suppressed stress. However, it should be noted that there is a difficulty to control the dosage of essential oil, since the main component of essential oils are volatile.

Atsumi and Tonosaki [86] observed that lavender and rosemary increase the free radical scavenging activity and decrease the cortisol level in saliva in humans. The effects of lavender aroma have been the most studied in the essential oils, but lavender-containing mixtures have also been investigated. For example, it was demonstrated that a mixture of lavender, ylang-ylang, marjoram, and neroli (20:15:10:2) suppresses the stress level and may have relaxation effects for controlling hypertension [104]. Aroma of limonene, a typical monoterpene contained in citrus fruits, has been also studied, since it is suggested that the aroma may be useful for human health [105]. Li et al. [99] examined the effects of essential oils from trees on male subjects who stayed at an urban hotel for three nights. The aromatic volatiles (phytoncides) were produced by vaporizing cypress (*Chamaecyparis obtusa*) stem oil with a humidifier in the hotel room during the night stay. The inhalation of the essential oil caused significant increases in the activity and proportion of natural killer (NK) cells in the blood. Decrements were found in the percentage of T cells and the concentrations of adrenaline and NA in the urine. Phytoncides, such as α-pinene and β-pinene, were detected in the air of hotel rooms. Therefore, phytoncide exposure may contribute to lowering stress hormone levels and increasing NK activity, which results in the enhancement of immune function.

Matsubara and Ohira [102] reported, in female subjects, that olfactory stimulation with the essential oil that is extracted from Japanese cedar (*Cryptomeria japonica*) wood may modulate mood states, and transiently suppress sympathetic nervous activity. Thus, the volatile compounds of the essential oil could contribute to mental health. One of the aliphatic aldehydes, *cis*-3-hexenol, has another name of green leaf aldehyde, together with the isomer *trans*-2-hexenal, and it is a major component of the smell of grass and leaf. It was demonstrated that a mixture of *cis*-3-hexenol and *trans*-2-hexenal has anti-fatigue effects, which suggests that the olfactory receptors in the olfactory system may be involved in the attenuation of fatigue [106]. Unique aromas have also been studied. Kunihiro et al. [108] demonstrated that volatile components in the essential oil of “Yomogi (*Artemisia montana*)” shows stress-reducing effects following nasal exposure. The sedative effects of yomogi oil is thought to be due to 1,8-cineol according to the near-infrared spectroscopy (NIRS) analysis. The inhalation of essential oil from legal hemp variety, in which the main components were myrcene and β-caryophyllene, affected the brain wave activity and autonomic nervous system, which suggested that the essential oil of the Cannabis has a neuro modular activity in cases of stress, depression, and anxiety [109].

As described above, stress-suppressing smells are well recognized, and essential oils are actually used for aromatherapy for stress reduction in humans [87]. Thus, the development of effective essential oil inhalers is underway [124]. However, the mechanism underlying the effect of aroma on the stress response in the brain has not yet been elucidated.

#### 3.3.2. Effect on Rats

Numerous investigators have revealed the stress-suppressing effect of essential oils, especially on the behavior of rodents [110]. First, let us look at previous studies in rats. It was demonstrated that the aroma of coffee beans reduced stress [111]. Hritcu et al. [88] reported the effect of lavender on spatial memory impairment by scopolamine. Thyme (*Tymus vulgaris*) [112] has the property of suppressing aflatoxin-induced oxidative stress. The essential oil from a lemongrass variety of *Cymbopogon flexuosus* (lemongrass oil) is used in various food and aroma industry products. Li et al. [113] demonstrated that lemongrass oil and its major component, citral, may affect the activities of drug-metabolizing enzymes and reduce the oxidative stress in the liver. Fukada et al. [91] reported that the destruction of the skin barrier due to chronic stress can be restricted or prevented by rose essential oil inhalation on the HPA axis in rats and human. Sadiki et al. [114] demonstrated that the essential oil of Tetraclinis (*Tetraclinis articulata*) could be a potent pharmacological agent against dementia by modulating the cholinergic activity and promoting antioxidant action in the rat hippocampus. Bergamot essential oil may act like an anxiolytic, similarly to diazepam, and reduce the corticosterone response to stress [96]. Behavioral studies combined with the measurement of *c-fos* expression in the rat amygdala suggested that vetiver essential oil may have the anxiolytic properties that might be associated with altering neuronal activation in the central amygdaloid nucleus [125]. It has been suggested that olfactory signals may mediate a phenomenon, called social buffering of conditioned fear responses in the rat [126]. The amygdala may play important roles in social buffering [55,126,127].

The smell of green, a mixture of *cis*-3-hexenol and *trans*-2-hexenal, can affect neuronal activity in the prelimbic and intralimbic of medial prefrontal cortex [73], which may have a preventive as well as a therapeutic effect on the depressive-like state [107]. These effects, at least in part, may be due to an up-regulation induced by the smell of green in the hippocampus. Park et al. [100] studied the effects of an essential oil from cypress on early life stress by means of maternal separation (MS) rats. Cypress inhalation decreased anxiety-related behavior in the elevated plus-maze test. The aroma also downregulated the expressions of cytokine genes, such as Cxcl10, Ccl19, and Il1rl, in addition to Ccl2 and Il6 in the hippocampus of MS rats. Thus, the aroma of cypress may attenuate MS-induced anxiety-related behaviors, and modulate cytokines, particularly Ccl2 and Il6, in the hippocampus.

Boiangiu et al. [92] examined the effects of an essential oil mix consisting of limonene (91.11%), γ-terpinene (2.02%), β-myrcene (1.92%), β-pinene (1.76%), α-pinene (1.01%), sabinene (0.67%), linalool (0.55%), cymene (0.53%), and valencene (0.43%) on the rat. This oil reversed scopolamine-induced memory deficits and oxidative stress in the brain, along with cholinesterase inhibitory effects, an important mechanism of anti-amnestic effects. These findings suggest that the inhalation of the essential oil mix can improve memory deficits through the activity of the cholinergic system and the restoration of antioxidant status in the brain.

#### 3.3.3. Effect on Mice

A growing body of evidence suggests the effects of aroma in the mouse, as follows. Matsukawa et al. [72] demonstrated, in mice, that the aroma of rose can counteract the odor of predators. Ueno et al. [128] suggested that 2-phenylethanol, the main aroma component of rose oil, elicits neuropsychological effects that alter the behavior of mice and may also elicit anti-depressive effects. Thus, the inhalation of rose oil containing 2-phenylethanol may be effective against depression and stress-related diseases. It was also demonstrated that by lavender [89,90], cypress [101], α-pinene [103], and thyme linalool suppressed stress [121,122]. It was Hashikawa-Hobara et al. [115] suggested that inhalation of roman chamomile (*Chamaemelum nobile*) may enhance the antidepressant effect of clomipramine by increasing hippocampal neurogenesis and regulating corticosterone levels in patients with treatment-resistant depression. Komiya et al. [116] showed a strong anti-stress effect of lemon essential oil by the behavior of mice. Their phenomenological results suggest that the antidepressant-like effects of lemon oil are closely associated with the 5-HTergic pathway, especially via the 5-HT (1A) receptor. They also found that lemon oil significantly accelerated the metabolic turnover of hippocampal DA and prefrontal cortex and striatal 5-HT.

The effects of woody (hinokitiol) aroma were examined in mice by plasma corticotropin levels, c-Fos immunoreactivity in the piriform cortex, and bed nucleus of stria terminalis, and it was suggested that hinokitiol ameliorates TMT-induced stress [117]. In phytotherapy, essential oils tend to be used daily for a period of days or weeks rather than a single application. Satou et al. [129] investigated the effects of the inhalation of α-pinene, an essential oil component, on the mouse behavior and accumulation in the brain and liver. Significant anxiolytic-like activity was consistently observed during the five-day inhalation of α-pinene. On the other hand, the accumulation of α-pinene in the brain and liver peaked on the third day of inhalation. Stress appears to affect the accumulation of α-pinene in the internal organs, keeping the anxiolytic-like action constant. Zhang et al. [118] investigated the effects of navel orange (*Citrus sinensis* (L.) Osbeck) essential oil and its main volatile component, limonene, on the mouse receiving chronic unpredictable mild stress. The essential oil inhalation significantly restored the depression-like behaviors and symptoms in the stressed mice. Limonene significantly improved depressive behavior, hyperactivity of the HPA axis, and decreased monoamine neurotransmitter levels, as well as suppressing the down-regulation of BDNF and its receptor expression in the hippocampus. These data suggest that limonene plays important roles in antidepressant effects.

#### 3.3.4. Biochemical Parameters in the Brain in Response to Olfaction

Regarding alterations in the expression of biochemical parameters in the brain to clarify the relationship between olfaction and stress, immediate-early genes have been frequently measured. Many of the immediate-early genes in neurons are “activity-dependent genes”, whose expression is induced by synaptic activity and the influx of calcium ions that are associated with action potentials [130]. Typical immediate-early genes in the brain include genes encoding transcriptional regulators, such as *c-fos* and *Egr-1*, and gene encoding synapse-related proteins, such as *Arc* and *Homer1a*/*Vesl-1s*. The mRNAs and protein products of these genes are widely used as markers of neural activity [131,132]. Indeed, numerous researchers reported an alteration in the expression of c-fos gene [16,21,59,68] and Arc gene [132]. c-Fos immunoreactivity was also analyzed in the rat amygdala [96], bed nucleus of the stria terminalis [57], and hypothalamus [74,126]. Kigar et al. [59] suggested that the methylation of adenosine in mammalian DNA can be used as an epigenetic biomarker for investigating the development of stress-induced neuropathology.

Exceptionally, studies on PTSD model rats showed changes in the expression of GABA_A_ receptor subunit α1 in the amygdala and hippocampus [133]. In other PTSD model rats by the odor of dirty cat litter, Aykac et al. [134] found alterations in the expression of muscarine receptors, M1, M2, M4, and M5 in the frontal cortex, hippocampus, and amygdala. Fluoxetine and propranolol ameliorated these changes in the frontal cortex. Ozbeyli et al. [135] suggested that, when vortioxetine is administered immediately after trauma, it may reduce anxiety, cognitive, and neuronal impairment, and it may prevent the development of PTSD by predator odor. Based on the action of vortioxetine, a possible decrease in 5-HTergic neurons is considered to be very important in the brain of PTSD. However, analysis of biochemical parameters in the brain, such as stress marker candidates, including neurotransmitters, proteins, peptides, etc., has not yet been sufficiently accumulated.

## 4. Recent Studies on the Effects of Aroma on Biochemical Parameters in the Brain

As described above, the effect of aroma on the stress response in the brain has not been well documented yet. The authors focused on the stress-suppressing effect of certain aroma in the process of analyzing the effect of stress on the brain. Conventionally, it is empirically recognized that a certain aroma has a stress-suppressing effect, although its effectiveness has not been fully proven [10]. To attempt a proof of the effect of aroma, we examined, in experimental animals, some kinds of smell by means of behavioral analysis and measuring biochemical parameters that have recently been identified as potential stress marker candidates in the brain and blood. Here, we would like to introduce our recent studies on the effects of aroma of coffee beans, lavender, cypress, α-pinene, and thyme linalool.

### 4.1. Coffee Beans

The debate about the health effects of coffee has not yet been settled, but the beneficial effects of drinking coffee are known to reduce the risk of developing depression and suicide [136] and relieve stress [137]. It should be noted that previous studies have primarily focused on the non-volatile component caffeine, whose awakening effect can be stressful. On the other hand, changes in the brain due to volatile components have not well been clarified. Interestingly, Tabuchi et al. [119] demonstrated that experimental animal rats prefer the aroma of coffee.

The authors [111] divided rats into four groups (control group, stress group, coffee group, and stress + coffee group) and the rats were bred in each environment for 24 h. For the stress and stress + coffee groups, a very mild water immersion stress (stress of putting water instead of wood chips for control and coffee groups) was applied. After that, the whole brain was removed, and the expression of genes and proteins was analyzed. As a result, it was assumed that alterations in the expression of some genes and proteins that are caused by stress was suppressed by the coffee bean aroma. Such stress-suppressing effects of the aroma was notably observed in the expression of *nerve growth factor receptor* (*NGFR*), *high affinity neurotrophic tyrosine kinase, receptor type 3* (*trkC*), *glucocorticoid-induced receptor* (*GIR*), and thiol-specific antioxidant protein and heat shock 70 kDa protein 5. Based on the annotation regarding the function of each factor, it was suggested that the coffee bean aroma might have antioxidant and stress-relieving functions.

For example, the nerve growth factor (NGF) is known to be resistant to oxidative stress and it promotes cell survival by activating free radical scavenging enzymes and glutathione peroxidase. The expression levels of NGFR gene were decreased in the stress group, and the stress + coffee group showed higher levels than that of the stress group, which suggests that the aroma of coffee beans suppressed oxidative stress induced by water immersion stress. GIR is a G protein-coupled receptor for which no ligand has been identified (orphan receptor). The GIR gene expression levels were also decreased due to stress, and the expression in the stress + coffee group was increased comparing to the stress group. Because GIR is involved in anxiety and neuroendocrine regulation, the aroma of coffee beans may have anxiolytic effects. Thiol-specific antioxidant proteins are known to suppress oxidative stress. The expression level in the stress + coffee group was higher than that in the stress group. These results suggest that the aroma of coffee beans may have an antioxidant effect. These factors in the brain were considered to be potential stress marker candidates. The roasted coffee bean aroma could change the mRNA and protein expression levels of the rat brain, providing clues as to the potential antioxidant or stress relaxation activities of volatile molecules of coffee beans. These data on the effects of volatile components of coffee beans are completely different from those investigating the effects of caffeine intake.

### 4.2. Lavender

Previous studies suggest that lavender has a stress-suppressing effect and it may be effective in preventing depression [89]. As the next step, the authors analyzed the effect of lavender (*Lavandula officinalis*) essential oil (LvEO) [90]. The mice were divided into four groups (control group, stress group, LvEO group, stress + LvEO group) and then bred in each environment for 24 h. As the stress, water immersion stress was applied as in the coffee bean aroma experiment [111]. Subsequently, distilled water or LvEO was inhaled by the mice for 90 min. An elevated plus-maze test, which is one of the evaluation methods for anxiety-related behavior, suggested that LvEO had anxiolytic effects. When LvEO was inhaled by the mice, the rate of entry into open arms and the time that was spent in open arms were increased significantly regardless of the presence or absence of stress. Moreover, the rate of entering and the time spent in open arms in the stress + LvEO group were significantly higher than those in the control group. Furthermore, the total distance that was traveled in the stress + LvEO group was significantly longer than that in the control group, indicating an increase in locomotor activity. These results strongly suggested that LvEO had anxiolytic activity.

**Figure 3:**
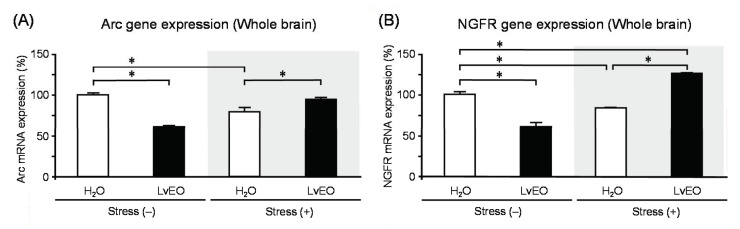
The effects of lavender on gene expression levels in the mouse brain. RT-PCR analysis was performed using the whole brain of mice inhaled with distilled water or lavender essential oil (LvEO) after water immersion stress. (**A**): Expression of Arc gene. (**B**): Expression of nerve growth factor receptor (NGFR) gene. The results are expressed as the ratio of the mRNA expression level to that of glyceraldehyde-3-phosphate dehydrogenase (GAPDH) (%), and are means ± SEM (n = 4). * *p* < 0.05 (Tukey–Kramer’s HSD test). This figure is modified based on the original figure published [90].

**Figure 4:**
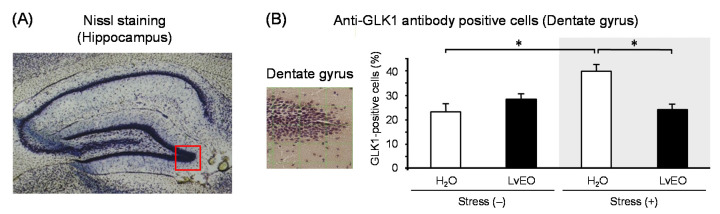
The effects of lavender on protein expression levels in the mouse brain. After water immersion stress, brain sections of mice inhaled with distilled water or LvEO were stained immunohistochemically with anti-galactokinase 1 (GLK1) antibody. (**A**): Nissl-stained mouse brain frontal section at the level of the hippocampus. (**B**): Expression of GLK1 positive cells in the dentate gyrus, inside the photo square indicated in (**A**). The results are expressed as the ratio of the number of GLK1 positive cells to that of Nissl-stained cells (%), and are means ± SEM (n = 4). * *p* < 0.05 (Tukey–Kramer’s HSD test). This figure is modified based on the original figure published [90].

In the whole brain, the expression level of Arc mRNA of the stress group and the LvEO group was significantly lower than that of the control group. On the other hand, the stress + LvEO group was equivalent to the control group level (Figure 3A). The gene expression of *NGFR* showed similar changes as *Arc*, but the stress + LvEO group showed a significant increase when compared to the control group (Figure 3B). These results suggested that LvEO had an anti-stress effect. In immunohistochemistry, the ratio of the number of positive cells in dentate gyrus of the hippocampus to the number of Nissl-stained cells was calculated. The expression level of galactokinase (GLK) 1 was significantly increased in the stress group and decreased to the same level in the stress + LvEO group as in the control group (Figure 4). Therefore, LvEO might have a sedative effect by suppressing the energy expenditure (hypermetabolism) due to arousal/insomnia.

The expression level of BDNF, which was regarded as a promising stress marker, tended to decrease in the order of LvEO group, stress group, and stress + LvEO group, but no significant change was observed in any of the groups. It might be possible that the stress level in this study was not the intensity that significantly reduced BDNF expression. Because BDNF expression is significantly reduced after the onset of depression, BDNF is considered to be an effective biomarker when stress causes serious brain damage. The results of behavioral analysis suggested that LvEO had anxiolytic activity and was accompanied by an increase in locomotor activity, as described above. From the results of expression analysis of genes and proteins in the brain, it was suggested that LvEO had an anti-stress effect, and it exerted a sedative effect. The relationship between sedation and increased locomotor activity should be further investigated. As a volatile component of lavender, the molecules of linalool and linalyl acetate may considered to affect the olfactory system.

Recently, Yoshida et al. [120] investigated gene expression profiles by means of DNA microarray analysis in the hypothalamus, and the found that inhalation of racemic (*R*,*S*) -linalool restored the expression of numerous stress-induced genes to normal. These genes were associated with synaptic transmission via neurotransmitters, including anxiolytic neuropeptides, such as oxytocin and neuropeptide Y, and it also contained several major histocompatibility complex (MHC) class I molecules that were required for neurodevelopment and plasticity. In addition, it was suggested that inhalation of (*R*,*S*) -linalool under stress might activate prolactin. These results suggested some of the molecular mechanisms associated with aroma inhalation in the hypothalamus of stressed organisms. 

### 4.3. Cypress

Cypress is a tree that is especially familiar to Japanese people and it has been used as a fragrance and building material for a long time. The authors investigated the effect of cypress aroma on the emotional behavior of mice and the expression level of stress marker candidates in the brain [101]. Rodents, such as mice and rats, are comfortable in an environment where multiple individuals are bred in the same cage, and single breeding is stressful. In this study, five-week-old male ICR mice were bred alone for one week (one animal/cage), and then each mouse was placed in a glass container and inhaled with cypress essential oil or distilled water for 90 min. Immediately after inhalation, an elevated plus-maze test was performed. After the behavioral test, the whole brain was removed, and the expression level of the stress marker candidates was measured. The gene expression of *NGFR* and *Arc* was analyzed by RT-PCR, and the protein expression of BDNF and GLK1 by immunohistochemistry.

As a result of the elevated plus-maze test, the rate of visiting open arms and time spent in the open arms of the group inhaled cypress essential oil (7.0 mg/L air) were significantly increased when compared with the control group (*p* < 0.05). Mice with low anxiety levels entered and stayed in open arms more often, which suggested that the aroma of cypress might reduce the anxiety levels. The expression levels of *Arc* and *NGFR* in the cypress group (7.0 mg/L air inhalation) were significantly higher than those in the control group (*p* < 0.05). Arc plays important roles in long-term potentiation and long-term memory, and NGFR is a receptor for NGF that is resistant to oxidative stress. Because the expression levels of these genes were decreased by stress, the present results of the gene expression in the brain also suggested that the cypress aroma might suppress stress level. In immunohistochemistry, the number of BDNF and GLK1-positive cells in the cypress group was not significantly different from that in the control group. Alterations in protein expression could take longer time than those in gene expression, so changes in expression due to the aroma could not be observed under the present experimental conditions. These results suggested that the aroma of cypress had anxiolytic and stress-suppressing effects.

### 4.4. α-Pinene

**Figure 5:**
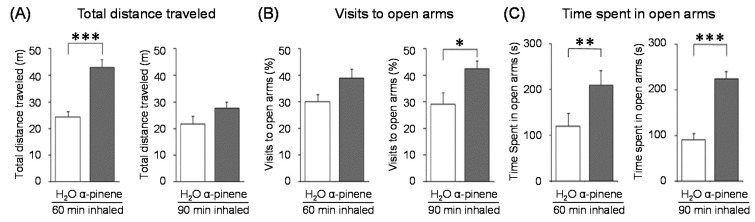
Effects of α-pinene on mouse behavior. Stressed mice were inhaled with distilled water or α-pinene for 60 or 90 min., and then an elevated plus-maze test was performed. (**A**): Total distance traveled (m). (**B**): Visits to open arms (%). (**C**): Time spent in open arms (sec). The results show means ± SEM (n = 5). * *p* < 0.05, ** *p* < 0.01, *** *p* < 0.001 (Student’s *t*-test). This figure is modified based on the original figure [103] with the copyright permission.

α-Pinene, which accounts for 50–60% of the cypress essential oil, is a pine-derived monoterpene, and it is the source of the unique aroma that is found in many conifers. The essential oils listed in descending order of α-pinene content are cypress, pine, rosemary verbenone, eucalyptus globulus, rosemary cineole, chamomile roman, and thyme linalool. The authors studied the effect of α-pinene on stressed mice [103]. After the mice were bred alone for one week, these animals were divided into four groups: 60 min. control group (distilled water inhalation for 60 min.), 60 min. α-pinene group (α-pinene inhalation for 60 min.), 90 min. control group (distilled water inhalation for 90 min.), 90 min. α-pinene group (α-pinene inhalation for 90 min.). After inhalation, an elevated plus-maze test was performed, and the total distance traveled was significantly increased in the 60 min. α-pinene group as compared to the 60 min. control group (Figure 5A). These results suggested that α-pinene might have an excitatory effect. The 90 min. α-pinene group showed a significant increase in the visits to open arms and the time spent in open arms, and the 60 min. α-pinene group caused a significant increase in the visits to open arms (Figure 5B,C), suggesting that α-pinene might have anxiolytic effects. However, the inhalation time of α-pinene, which caused significant changes in the excitatory and anxiolytic effects, did not match between 60 min. and 90 min. Therefore, we investigated the transfer of α-pinene into the brain. As a result of measuring the α-pinene concentration in the mouse brain after inhalation, the brain transferability by 60-min. inhalation was significantly higher than that by 90-min. inhalation. Thus, the brain transferability of α-pinene was not proportional to inhalation time, which suggested that the effect of α-pinene was due to both olfactory stimulation and transfer to the brain.

**Figure 6:**
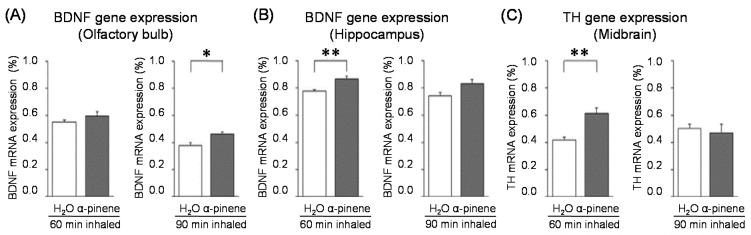
Effects of α-pinene on gene expression levels in the brain. After inhaling distilled water or α-pinene into stressed mice, the olfactory bulb, hippocampus, and midbrain were used for RT-PCR analysis. (**A**): Expression of BDNF gene in the olfactory bulb. (**B**): Expression of BDNF gene in the hippocampus. (**C**): Expression of tyrosine hydroxylase (TH) gene in the midbrain. The results are expressed as the ratio of the mRNA expression level of BDNF or TH to that of GAPDH (%) and are means ± SEM (n = 5). * *p* < 0.05, ** *p* < 0.01 (Student’s *t*-test). This figure is modified based on the original figure [103] with the copyright permission.

For genes in the brain regions, the BDNF gene expression level was significantly increased in the olfactory bulb of the 90-min. α-pinene group and the hippocampus of the 60-min α-pinene group (Figure 6A, B). Therefore, BDNF genes in the olfactory bulb and hippocampus might be involved in the anxiolytic effect of α-pinene. Nevertheless, the inhalation time of α-pinene, which caused a significant increase in BDNF gene expression, was different between the olfactory bulb and hippocampus. The effects of inhaled α-pinene might differ in time, depending on the brain region, and such a time lag should be clarified in the future. As mentioned above, behavioral analyses revealed that α-pinene had an excitatory-like effect. Therefore, we measured tyrosine hydroxylase (TH), a rate-limiting enzyme for the synthesis of catecholamines, gene expression in the midbrain. α-Pinene caused a significant augment in the *TH* expression level in the 60-min. α-pinene group (Figure 6C). These results suggested that α-pinene might activate DA neurons, which results in hyperactivities. On the other hand, α-pinene did not cause significant changes in the expression of TH gene in the medulla oblongata, suggesting that the NA neurons in this region were not significantly affected.

Although the amount of α-pinene transferred to the brain after inhalation for 90 min. was lower than that inhaled for 60 min., anxiolytic activity was observed. These results suggest that α-pinene and its metabolites might stimulate the olfactory system. An excitatory effect was observed after 60-min. inhalation, and an anxiolytic effect was observed after 90-min. inhalation. Therefore, the biological effects of volatile components could be mediated by both olfactory stimulation and the transfer of aroma molecules to the brain.

### 4.5. Thyme Linalool

Stress is thought to lead to anxiety and fatigue. Various mechanisms may be involved in fatigue, but brain fatigue has recently been of particular interest. Brain fatigue is caused by inflammation that occurs in the brain and affects the whole body. In the process of studying the effects of aroma on the brain, we have come to aim to scientifically prove the anti-fatigue effect of aromatherapy. We investigated the effect of thyme linalool on brain fatigue model animals [121]. There are a variety of types of thyme, with the most general one being common thyme (*Thymus vulgaris*), and one of the chemotypes is thyme linalool (*Thymus vulgaris* ct. linalool). Thyme linalool is an essential oil containing 51.2% linalool and 14.0% terpinen-4-ol. We studied the effect of thyme linalool (thyme). Mice received intraperitoneal (i.p.) administration of polyinosinic:polycytidylic acid (poly I:C) (20 mg/kg) as a brain fatigue model. Locomotor activities were measured using a rotating cage after the inhalation (i.h.) of thyme or distilled water with 2 μL/L air. The effect of thyme i.h on inflammatory cytokines was also analyzed.

**Figure 7:**
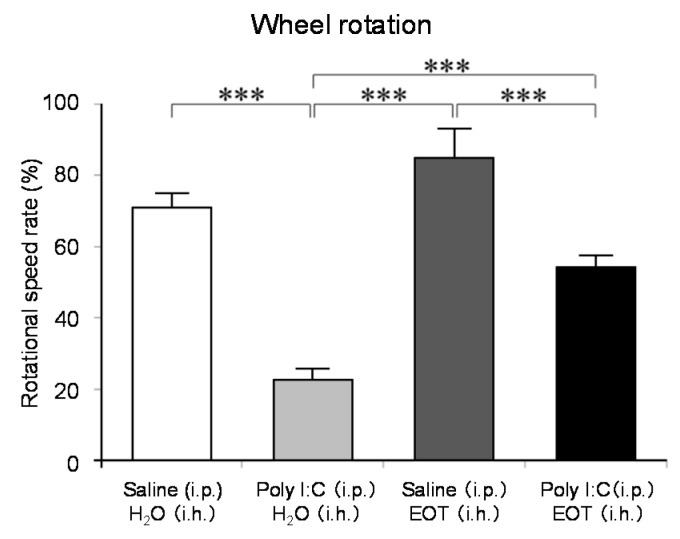
The effects of thyme on mouse activity. A brain fatigue model was prepared by intraperitoneal (i.p.) administration of poly I:C (10 mg/kg). Saline was similarly administered to the control group. After inhalation (i.h.) of thyme (EOT) (2 mL/L air) or distilled water, mice were examined by the wheel rotation test for 15 h. The results are expressed as the rotation speed rate (%) and are means ± SEM (n = 8). *** *p* < 0.001 (Tukey–Kramer test). This figure is modified based on the original figure [121] with the copyright permission.

**Figure 8:**
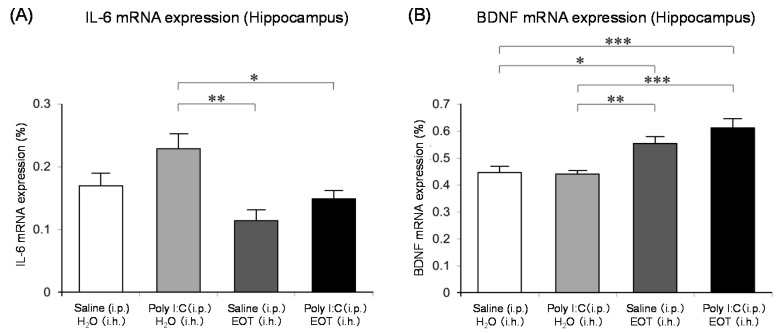
Effects of thyme on gene expression levels in the brain. Mice received i.p. injection of Poly I:C (20 mg/kg) or saline. After i.h. of thyme (EOT) (2 mL/L air) or distilled water, hippocampus was used for RT-PCR analysis. (**A**): Interleukin-6 (IL-6) gene expression in the hippocampus. (**B**): BDNF gene expression in the hippocampus. The results are expressed as the ratio of mRNA expression level of IL-6 or BDNF to that of GAPDH (%), and are means ± SEM (n = 5–7). * *p* < 0.05, ** *p* < 0.01, *** *p* < 0.001 (Tukey–Kramer test). This figure is modified based on the original figure [121] with the copyright permission.

The locomotor activity was significantly increased in the thyme i.h. group as compared with the control (Poly (I:C), i.p./distilled water, i.h.) group (Figure 7). These data suggested the anti-fatigue effect of thyme. The inhalation of thyme significantly reduced the mRNA expression level of interleukin-6, one of the inflammatory cytokines, in the hippocampus (Figure 8A). The expression level of BDNF could have been reduced in the hippocampus of stressed animals and the blood of patients with depression [15]. However, BDNF mRNA expression levels in the hippocampus were significantly increased in the thyme i.h group than the control group in the present study (Figure 8B).

**Figure 9:**
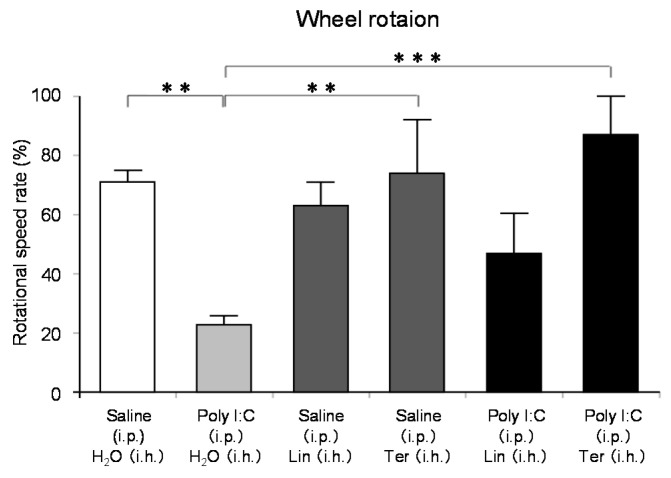
Effects of thyme components, linalool and (+)-terpinen-4-ol, on mouse behavior. A brain fatigue model mouse was prepared by i.p. injection of Poly I:C (20 mg/kg) or saline. After i.h. administration of (−)-linalool or (+)-terpinen-4-ol, mice were examined by the wheel rotation test for 15 h. The results are expressed as the rotation speed rate (%) and are means ± SEM (n = 4–8). Lin: (−)-Linalool (2 mL/L air), Ter: (+)-Terpinen-4-ol (2 mL/L air). ** *p* < 0.01, *** *p* < 0.001 (Tukey–Kramer test). This figure is modified based on the original figure [121] with the copyright permission.

The anti-fatigue effects of linalool and (+)-terpinen-4-ol, which are the main components of the essential oil used in this study, were also examined. The inhalation of (+)-terpinen-4-ol (2 μL/L air) caused a significant increase in locomotor activity of the brain fatigue model mice in comparison with the control group (Figure 9). The linalool-administered group showed a tendency of increase in locomotor activity, which was not statistically significant. The concentration of linalool in the brain was more than 10 times the (+)-terpinen-4-ol concentration. These results suggested that the inhalation of thyme might lead to anti-fatigue effects through anti-inflammatory effects and activating neurons. In this study, it was unclear whether the anti-fatigue effect of thyme was due to linalool or (+)-terpinen-4-ol, although it could not be denied that both of the molecules might be involved in the anti-fatigue effect.

**Figure 10:**
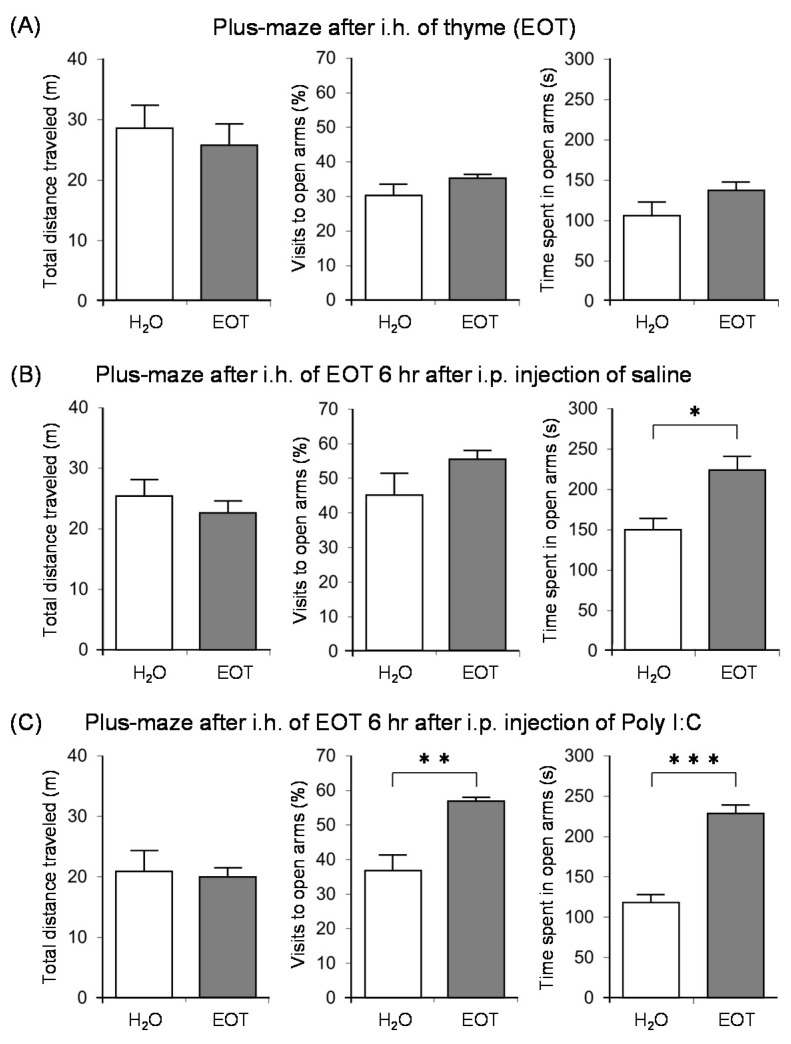
The effects of thyme on mouse behavior. (**A**): Elevated plus-maze test immediately after i.h. of thyme (EOT) (2 mL/L air) to mice bred alone for seven days. (**B**): Elevated plus-maze test immediately after i.h. of thyme (EOT) (2 mL/L air) 6 h after i.p. administration of saline to mice bred alone for seven days. (**C**): Elevated plus-maze test immediately after i.h. of thyme (EOT) (2 mL/L air) 6 h after i.p. administration of Poly I: C to mice bred alone for seven days. The results show means ± SEM (n = 5). * *p* < 0.05, ** *p* < 0.01, *** *p* < 0.001 (Student’s *t*-test). This figure is modified based on the original figure [122] with the copyright permission.

Subsequently, we investigated the anxiolytic effect of thyme with the aim of clarifying the relationship between anxiety, fatigue, and stress [122]. Male ICR mice were bred alone and divided into three groups: no treatment and i.p. administration of saline or poly I:C. After inhaling thyme for 90 min., the anxiety-related behavior was assessed by an elevated plus-maze test. The inhalation of thyme did not cause anxiolytic effects in mice with no treatment (Figure 10A). On the other hand, thyme showed significant anxiolytic effects in brain fatigue model mice with poly I:C. Indeed, the rate of visits to open arms and time spent in open arms were significantly increased by i.h. of thyme in the brain fatigue model mice (Figure 10C). Thyme also caused a significant increase in the time that was spent in open arms in mice with i.p. administration of saline (Figure 10B).

### 4.6. Transfer of Fragrant Molecules to the Brain

The biological effects of fragrant molecules can include not only the olfactory pathway, but also the blood flow due to nasal and transdermal absorption of volatile molecules. Monoterpenes are the main volatile component of plant essential oils. Olfactory receptors may recognize monoterpenes as smell and affect emotions. It is possible that the components of the essential oil act directly on the central nervous system as well as on the olfactory pathway. Terpenes have been known to cross the blood-brain barrier and have proven to interact with potential receptors, such as cannabinoid receptor type 1 [138]. However, the transferability of monoterpenes after inhalation, the main method of administration of essential oils in aromatherapy, has not been well clarified.

**Figure 11:**
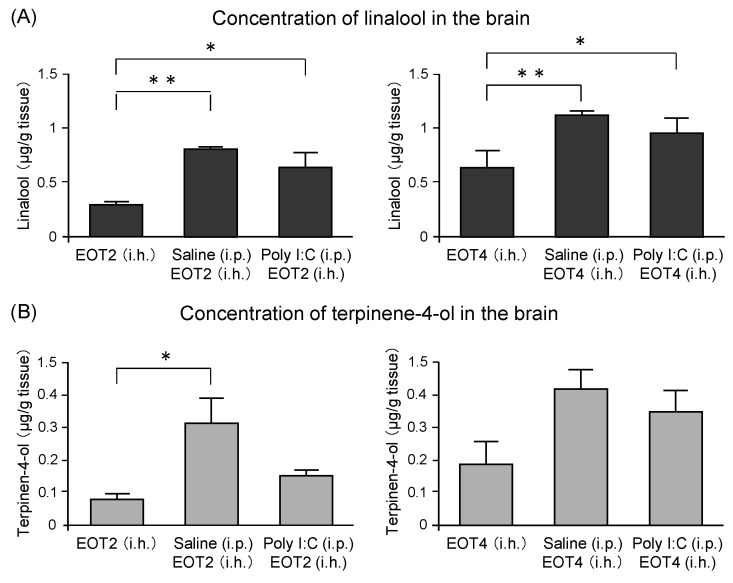
The concentrations of linalool and terpinen-4-ol in the mouse brain after i.h. of thyme. Three groups of mice loaded with weak stress by breeding alone: thyme (EOT) (i.h.) group, EOT (i.h.) after moderate stress by i.p. injection of saline or Poly I:C. (**A**): Linalool concentration after EOT (2 or 4 mL/L air, i.h.), (**B**): Terpinen-4-ol concentration after EOT (2 or 4 mL/L air, i.h.). The results show means ± SEM (n = 4). * *p* < 0.05, ** *p* < 0.01 (Tukey–Kramer test). This figure is modified based on the original figure [122] with the copyright permission.

The components of thyme that were transferred into the brain after thyme inhalation were analyzed by gas chromatography-mass spectrometry (GC/MS) [122]. Concentrations of linalool in the brain of the poly I:C group were significantly higher than those of mice inhaled with thyme alone (Figure 11A). An increment in the linalool concentration was also observed in mice that were treated with saline (Figure 11A). Terpinene-4-ol levels in the brain showed changes similar to those of linalool. A significant increase of terpinene-4-ol level was found in the saline group inhaled with thyme 2 mL/L air. An increasing tendency was observed in the saline group inhaled with thyme 4 mL/L air, which was not statistically significant (Figure 11B). These results suggested that stress and inflammation might disrupt the blood-brain barrier and, as a result, the anxiolytic and anti-fatigue effects of thyme could be enhanced. Further analysis was required on the mechanism by which the amount of volatile component transported to the brain by mechanical stressors, such as i.p. administration.

The authors also investigated the brain migration of (+)-α-pinene, (+)-limonene, (−)-linalool, and 1,8-cineole, which are the major monoterpenes of plant essential oils [139]. After inhalation of each compound into mice, the brain was removed, and each molecule was analyzed by GC/MS. As a result, it was clarified that α-pinene maximized brain transferability when inhaled for 30 min. This might be due to the high volatility of α-pinene. Limonene and linalool were maximally translocated to the brain after 90 min. of inhalation. The amount of linalool that was transferred to the brain was less than that of α-pinene and limonene. Brain transfer of 1,8-cineole was at its lowest level after 30 min. of inhalation. Furthermore, 1,8-cineole was easily transferred to the brain after i.p. administration. These results might be indicators for maximizing the effects of essential oils in aromatherapy. The transfer of volatile components of aroma to the brain needs to be clarified in the future. Under such circumstances, Aponso et al. [110] recently studied the identification of physicochemical properties that affect the volatility of essential oils and pathways of brain uptake by inhalation. The pharmacokinetics of volatile components are a major challenge in clarifying the effects of aroma on the brain.

For investigations on the effects of smell, it cannot be avoided that volatile components may affect organisms not only by the olfactory pathway, but also by other pathways, such as nasal and transdermal absorption. It should also be considered that even stress-reducing fragrant molecules can be toxic [140]. For example, terpenes, important components of essential oils, have been suggested to have hepatotoxicity. Because terpenes have been shown to penetrate the blood-brain barrier, it may be possible that the molecules have toxic effects in the brain. Intensive research on metabolism and the toxicity of terpenes is required to reduce the risk of injury from terpene-containing products, including essential oils [141].

## 5. Conclusions

This review briefly described the stress response of the brain and provided information on the biochemical parameters of the brain. From such a point of view, we summarized the literature on the relationship between the smell and stress response in the brain. The effects of stress on the brain and the pathogenic mechanism of mental disorders are being elucidated. Research on stress markers have continued to progress and the understanding of brain function is further evolving. It is interesting that the effect of smell on stress response in the brain is becoming clarified. Most of the previous studies have shown that a certain aroma can suppresses stress levels, and neuropharmacological and neurochemical studies are providing scientific evidence of the stress-suppressing effects of the smell. On the other hand, the smell also has stress-enhancing effects. The investigation of the relationship between the smell and stress response in the brain requires multifaceted analysis in the future. From this point of view, it is necessary to devise ways to accurately control the dose of volatile components. Elucidating the mechanisms of stress suppression by the smell will deepen the comprehension of living organisms and enable the effective use of volatile components. Multifaceted research on volatile molecules may contribute not only to aromatherapy, but also to food development. Indeed, we recently demonstrated the effect of sesame oil on mouse behavior and the expression of stress marker candidates [123]. In the future, it will be necessary to link chemical research at the level of molecules with neuropharmacology and neurochemistry.

## Figures and Tables

**Table 1:** Summary of smell and its main component(s) in previous studies. The scientific name of plants and the name of main component(s) are as described in the reference. A general name appears in the case of no indication in the article.

Smell	Scientific Name	Main Component(s)	References		
			Human	Rat	Mouse
Many kinds of aroma, such as lavender, rosemary, bitter orange, peppermint, linalool, limonene			[4] (Review)		
Partner smell			[9]		
Black tea		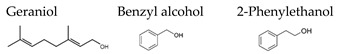	[81]		
Lavender	*Lavandula*	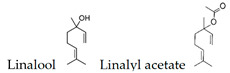	[82,83,84,85,86,87]	[88]	[89,90]
Chamomile	*Matricaria recutita*	Chamizulene 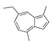	[83]		
Rosemary	*Rosmarinus officinalis*	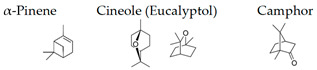	[86]		
Rose	*Rosa*	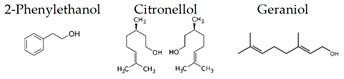	[91]	[91]	[72]
2-Phenylethanol		2-Phenylethanol 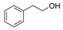			[92]
Bergamot	*Citrus bergamia*	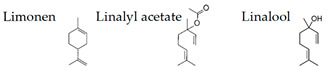	[93,94,95]	[96]	
Grapefruit	*Citrus paradisi*	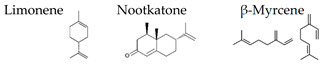	[97]		
Neroli	*Citrus aurantium* L. var. *amara*	Linalool 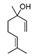	[98]		
Cypress	*Chamaecyparis obtusa*	α-Pinene 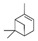	[99]	[100]	[101]
Cedar	*Cryptomeria japonica*	α-Pinene 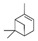	[102]		
α-Pinene		α-Pinene 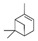			[103]
Mixture of essential oils			[104]		
Lavender	*Lavandula officinalis*	Linalool 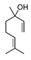 Linalyl acetate 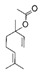			
Ylang-ylang	*Cananga odorata*	Germacrene D 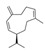			
Majoram	*Origanum majorana*	Terpinen-4-ol 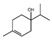 γ-Terpinene 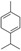			
Neroli	*Citrus aurantium*	Linalool 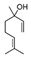			
Smell	Scientific name	Main component(s)	References		
			Human	Rat	Mouse
Limonene		Limonene 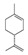	[105]		
Green		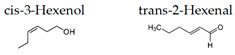	[106]	[73,107]	
Yomogi	*Artemisia montana*	Cineol (Eucalyptol) 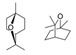	[108]		
Cannabis	*Cannabis sativa*	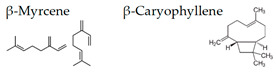	[109]		
Many kinds of volitile components				[110]	
Coffee bean	*Coffea*	Furfuryl alcohol 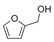		[111]	
Thyme	*Tymus vulgaris*	Carvacrol 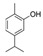 Thymol 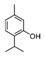		[112]	
Lemongrass	*Cymbopogon flexuosus*	Citral 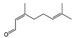		[113]	
Tetraclinis	*Tetraclinis articulata*	Hinokitiol 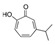 α-Pinene 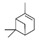		[114]	
Mixture of essential oil components		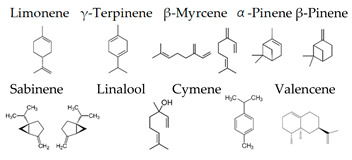		[92]	
Roman chamomile	*Chamaemelum nobile*	Angeric acid 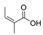			[115]
Lemon	*Citrus limon*	Limonene 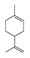			[116]
Hinokitiol		Hinokitiol 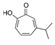			[117]
Navel orange	*Citrus sinensis* (L.) Osbeck	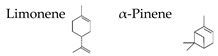			[118]
Thyme linalool	*Thymus vulgaris* ct. linalool	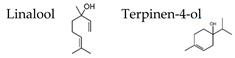	[119,120]		[121,122]
Sesame oil		2-Methylpyrazine 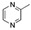		[123]	

## Data Availability

Not applicable to this article.
